# Antioxidant Effects of DPP-4 Inhibitors in Early Stages of Experimental Diabetic Retinopathy

**DOI:** 10.3390/antiox11071418

**Published:** 2022-07-21

**Authors:** Hugo Ramos, Patricia Bogdanov, Jordi Huerta, Anna Deàs-Just, Cristina Hernández, Rafael Simó

**Affiliations:** 1 Diabetes and Metabolism Research Unit, Vall d’Hebron Research Institute (VHIR), 08035 Barcelona, Spain; hugo.ramos@vhir.org (H.R.); patricia.bogdanov@vhir.org (P.B.); jordi.huerta@vhir.org (J.H.); anna.deas@vhir.org (A.D.-J.); 2 Center for Networked Biomedical Research of Diabetes and Associated Metabolic Diseases (CIBERDEM), Carlos III Health Institute (ICSIII), 28029 Madrid, Spain; 3 Department of Medicine, Autonomous University of Barcelona, 08193 Barcelona, Spain

**Keywords:** diabetes, diabetic retinopathy, sitagliptin, dipeptidyl peptidase-4, DPP-4 inhibitors, oxidative stress, antioxidant, NRF2

## Abstract

Hyperglycemia-induced oxidative stress plays a key role in the impairment of the retinal neurovascular unit, an early event in the pathogenesis of DR. The aim of this study was to assess the antioxidant properties of topical administration (eye drops) of sitagliptin in the diabetic retina. For this purpose, db/db mice received sitagliptin or vehicle eye drops twice per day for two weeks. Age-matched db/+ mice were used as the control group. We evaluated retinal mRNA (RT-PCR) and protein levels (Western blotting and immunohistochemistry) of different components from both the antioxidant system (NRF2, CAT, GPX, GR, CuZnSOD, and MnSOD) and the prooxidant machinery (PKC and TXNIP). We also studied superoxide levels (dihydroethidium staining) and oxidative damage to DNA/RNA (8-hydroxyguanosine immunostaining) and proteins (nitrotyrosine immunostaining). Finally, NF-кB translocation and IL-1β production were assessed through Western blotting and/or immunohistochemistry. We found that sitagliptin protected against diabetes-induced oxidative stress by reducing superoxide, TXNIP, PKC, and DNA/RNA/protein oxidative damage, and it prevented the downregulation of NRF2 and antioxidant enzymes, with the exception of catalase. Sitagliptin also exerted anti-inflammatory effects, avoiding both NF-кB translocation and IL-1β production. Sitagliptin prevents the diabetes-induced imbalance between ROS production and antioxidant defenses that occurs in diabetic retinas.

## 1. Introduction

Oxidative stress is defined as the imbalance between the formation and accumulation of reactive oxygen species (ROS) and the capacity of a biological system to neutralize them [1]. ROS as superoxide anion (O_2_^•^^−^), peroxyl radical (ROO^•^), and reactive hydroxyl radical (^•^OH) are molecular species with one or more unpaired electrons that are consequently unstable, highly reactive, and can lead to protein, lipid, and DNA damage at high concentrations [2]. These products are produced physiologically and are necessary for the proper functioning of multiple cellular processes. The maintenance of physiological and non-damaging levels of ROS is regulated by antioxidant defenses [3]. Overproduction of ROS or defects in the antioxidant machinery can result in oxidative stress, which is strongly implicated in the development of several diseases, such as diabetes and its complications [4].

Diabetic retinopathy (DR) is one of the most frequent complications of diabetes and the leading cause of preventable blindness among the work-aged population in high-income countries [5]. DR is currently considered a neurovascular complication, as hyperglycemia and its associated metabolic pathways cause not only microvascular damage but also neurodegeneration and impairment of the retinal neurovascular unit (NVU) [6]. This unit is an interdependent and functional coupling between neurons, glial cells, and blood vessels that coordinates vascular flow with metabolic activity. Any disruption in this functional unit may result in deleterious consequences, resulting in a structural and functional impairment of microvasculature and neurons [7]. NVU impairment is an early event in the pathogenesis of DR, and one of the major triggers is oxidative stress. It should be noted that the retina has a high metabolic and high oxygen-consuming rate, and therefore has special susceptibility to oxidative events [8]. The NVU is equipped with powerful antioxidant mechanisms, including nonenzymatic and enzymatic antioxidants to neutralize ROS and a repair system for those molecules that have been already oxidized [9]. Nevertheless, chronic hyperglycemia leads to excessive accumulation of ROS and reduces the activity and efficiency of these antioxidant defenses, enhancing ROS production [4]. Little is known about how exactly hyperglycemia induces these abnormalities, but four classical pathways have been highly related: the augmented influx of glucose by polyol and hexosamine pathways, the activation of protein kinase C (PKC) and the formation of advanced glycation end products (AGEs) [4,10]. The consequences of hyperglycemia-induced oxidative stress in the retina are mitochondrial dysfunction, inflammation, cellular apoptosis, and structural and functional modifications that consequently may result in neurodegeneration and early microvascular impairment [10].

Current treatments for DR are indicated only in advanced stages of the disease, and are expensive, invasive, and associated with severe side effects. Therefore, the search for new treatments targeting the earliest and asymptomatic stages of DR at the onset of NVU impairment is an unmet medical need that has to be urgently addressed [11]. In this context, a new therapeutic strategy is the topical administration (eye drops) of dipeptidyl peptidase-4 (DPP-4) inhibitors (DPP-4i), which have demonstrated promising results in experimental models of DR [12,13]. Through the nondegradation of glucagon-like peptide-1 (GLP-1), the main target of the DPP-4 enzyme, and through other independent and not clearly elucidated mechanisms, DPP-4i significantly prevents the appearance of the main hallmarks of DR, glial activation, and neuronal cell death, as well as preserving the functionality of the retina [12,13,14]. In the present study, we explored the potential antioxidant effects of topical administration of DPP-4i in an experimental model of DR.

## 2. Materials and Methods

### 2.1. Animals

Twenty diabetic male db/db (BKS.Cg-Dock7m +/+ Leprdb/J) mice and 10 nondiabetic mice (db/+; (BKS.Cg-Dock7m + Leprdb/+) were acquired from Charles River Laboratories (Calco, Italy) at 7 weeks of age for the experiments. The mutated leptin receptor carried by db/db mice gave rise to an obesity-induced type 2 diabetes phenotype. Mice were bred and maintained in the animal facilities of the Vall d’Hebrón Research Institute (VHIR). With the aim of minimizing variability, mice were randomly distributed (block randomization) into groups of two mice per cage in Tecniplast GM-500 cages (36 cm × 19 cm × 13.5 cm) under standard laboratory conditions at 22 ± 2 °C, with relative humidity of 50–60% and a 12 h light/dark cycle. The cages were equipped with nesting material, absorbent bedding (BioFresh Performance Bedding 1/800 Pelleted Cellulose, Absorption Corp, Ferndale, WA, USA), ad libitum food (ENVIGO Global Diet Complete Feed for Rodents, Mucedola, Milan, Italy), and filtered water. Glycemia was measured weekly through tail-vein blood sampling and a blood glucose meter (71371-80, FreeStyle Optium Neo; Abbott, IL, USA).

All animal experiments were directed in agreement with the European Community (86/609/CEE) and the guidelines of the Association for Research in Vision and Ophthalmology (ARVO) for the utilization of laboratory animals. The Animal Care and Use Committee of VHIR (CEEA 14/21) authorized the present study.

### 2.2. Topical Ocular Treatment

Db/db mice aged 10 weeks received a topical ocular administration of 10 mg/mL sitagliptin phosphate monohydrate (Y0001812, Merck KGaA, Darmstadt, Germany) or vehicle eye drops [phosphate buffered saline (PBS)] for 2 weeks twice per day. Eye drops were randomly administered with the aid of a micropipette (5 µL), onto the superior corneal surfaces of both eyes of diabetic mice. On day 15, 1 h before euthanasia, animals received an additional dose of each treatment. Db/+ mice matched by age were used as the control group.

### 2.3. Retinal Tissue Processing

On day 15, mice were intraperitoneally injected with 200 µL of anesthesia, composed of a mix of ketamine (1 mL) (GmbH, Hameln, Germany) and xylazine (0.3 mL) (Laboratorios Calier S.A., Barcelona, Spain). Once anesthetized, animals that were intended to be used for immunofluorescence experiments were transcardially perfused with 4% paraformaldehyde (sc-281692, Santa Cruz Biotechnology, Dallas, TX, USA), while others were euthanized through cervical dislocation. Ocular globes were rapidly enucleated and differently processed depending on their purpose. All eyes, with the exception of those used for the immunofluorescence experiment, were dissected and the retinas obtained. Six retinas from each experimental group were submerged in 140 µL of TRIzol reagent (15596018, Invitrogen, Carlsbad, CA, USA) and assigned in different tubes until RNA extraction. Another six retinas from each group were immediately distributed in empty and distinct tubes until protein extraction. All of them were stored at −80 °C. Finally, the retinas from four animals of each group were not obtained after enucleation, and the entire ocular globes were fixed again in 4% paraformaldehyde for 5 h before paraffin embedding.

### 2.4. RNA Isolation and Quantitative Reverse Transcription Polymerase Chain Reaction (RT-PCR) Assay

Retinas (stored at −80 °C in 140 µL of TRIzol) were treated with DNase (18068015, ThermoFisher Scientific, Waltham, MA, USA) to avoid genomic contamination and were purified on an RNeasy MinElute column (74106, Qiagen, Hilden, Germany). After supernatant removal, RNA sediment was obtained and resuspended in 30 µL of RNAse free water (AM9937, ThermoFisher Scientific, Waltham, MA, USA). A Nanodrop spectrophotometer and an Agilent 2100 Bioanalyzer and were used for both RNA quantification and integrity, respectively. Reverse transcription of cDNA was assessed using Oligo(dT)18 Primers (SO131, ThermoFisher Scientific, Waltham, MA, USA) and a High-Capacity cDNA Reverse Transcription Kit (4368814, ThermoFisher Scientific, Waltham, MA, USA) and with the help of a T100 Thermal Cycler (Bio-Rad, Hercules, CA, USA). RT-PCR was performed using SYBR Green PCR Master Mix (04707516001 Roche Diagnostics, Mannheim, Germany), specific primers (Table 1), a LightCycler480 System (05015243001, Roche Diagnostics, Mannheim, Germany), and 384-well optical plates (04729749001, Roche Diagnostics, Mannheim, Germany). Relative quantifications were obtained using the LightCycler480 SW 1.5.1 software and displayed as fold change versus control mice. *B2m* and *Actin* were used as housekeeping genes.

### 2.5. Western Blotting

Ten to fifteen seconds of sonication in 80 µL of lysis buffer [phenylmethanesulfonylfluoride (PMSF), 1 mM; Na3VO4 2 mM; NaF, 100 mM; 1× protease inhibitor cocktail (P8340, Sigma-Aldrich, St. Louis, MO, USA); and RIPA buffer (R0278, Sigma-Aldrich, St. Louis, MO, USA)] were used for retinal protein extraction. Extracted protein (25 µg) was loaded in 4–20% (*v/v*) mini-PROTEAN TGX precast protein gels (4561096, Bio-rad, Hercules, CA, USA). Electrophoresis was carried out for 90 min at 100 V. Proteins were then transferred from gradient gels to a polyvinylidene difluoride (PVDF) membrane (1620177, Bio-Rad Laboratories, Hercules, CA, USA) for 90 min at 400 mA. Membranes were then blocked in 5% bovine serum albumin (A3059-100G, Sigma-Aldrich, St. Louis, MO, USA) or 5% powdered milk (Central Lechera Asturiana, Spain). Both blocking solutions were prepared in 0.1% TBS-Tween. Primary antibodies (Table 2) were applied overnight at 4 °C. Secondary antibodies [goat anti-rabbit and goat anti-mouse (Dako Agilent, Santa Clara, CA, USA)] were incubated (1:10,000). Immunoreactive bands were acquired using a WesternBright ECL HRP substrate kit (K-12045-D50, Advansta, CA, USA) and quantified with Image J software (version 1.53. U. S. National Institutes of Health, Bethesda, MD, USA). Vinculin protein was assigned as loading control to normalize protein expression.

### 2.6. Immunofluorescence Analysis

Eyes were embedded in paraffin, then sectioned (3 µm) and mounted on 25.5 mm × 75.5 mm × 1.0 mm poly-*_L_*-lysine positively charged slides (S21.2113.A, Leica Biosystems, Wetzlar, Germany). For sample deparaffinization and rehydration, slides were consecutively submerged in 100% xylene (3 × 5 min), 100% ethanol (1 × 5 min), 96% ethanol (1 × 5 min), 70% ethanol (1 × 5 min), 50% ethanol (1 × 5 min), and distilled water (1 × 5 min). Samples were fixed again in ice-cold acid methanol (−20 °C) for 1 min and washed 3 times with PBS 0.01 M pH 7.4. For antigen retrieval, slides were warmed with a pressure cooker at 150 °C for 4 min in 250 mL of 1:10 diluted antigen retrieval solution with sodium citrate 10 mM, pH 6 (ab973, Abcam, Cambridge, UK). After 3 washes with PBS, sectioned eyes were incubated in blocking solution (X0909, Dako Agilent, Santa Clara, CA, USA) for 1 h at room temperature to avoid aspecific binding. Consecutively, specific primary antibodies (Table 3) were applied nightly at 4 °C. Next morning, after 3 washes in PBS, slides were incubated for 1 h in the dark with secondary antibodies (Table 3). Eye sections were washed one more time with PBS, counterstained with Hoechst 33342 (bisbenzimide) (14533, ThermoFisher Scientific, Waltham, MA, USA), and finally mounted with a coverslip (15747592, ThermoFisher Scientific, Waltham, MA, USA) and prolonged mounting medium fluorescence (P36930, Prolong, Invitrogen^TM^, Thermo Fisher Scientific, Eugene, OR, USA). A laser confocal microscope (Fluoview FV1000 Laser Scanning Confocal Microscope Olympus, Hamburg, Germany) was used for image acquisition (1024 × 1024 pixels). For detecting superoxide levels, dihydroethidium was applied as a primary antibody, and after three washes in PBS was counterstained with Hoescht and mounted.

### 2.7. Statistical Analysis

Graph bars are displayed as the mean value followed by the standard error of the mean (SEM). Means of the different experimental groups were compared using both Students *t*-test and one-way ANOVA, which was accompanied by Bonferroni multiple-comparison post hoc test. Differences were considered statistically significant when *p* < 0.05.

## 3. Results

### 3.1. Body Weight and Systemic Blood Glucose Levels of db/db Mice Not Modified after Topical Ocular Administration of Sitagliptin

**Figure 1:**
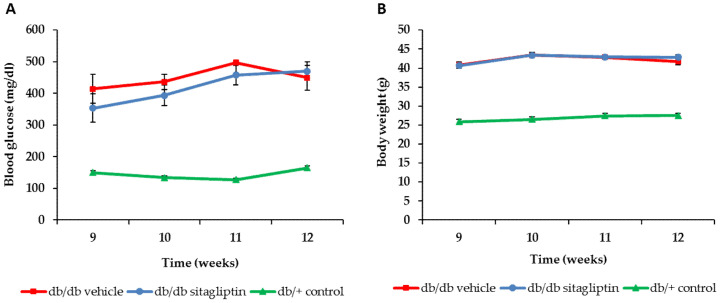
Evaluation of body weight and systemic blood glucose levels. (**A**) Blood glucose measurements during the experimental course of db/db mice treated with sitagliptin (blue circles) or vehicle (red squares) and db/+ mice (green triangles); *n* = 10. (**B**) Body weight course of db/db mice treated with sitagliptin (blue circles) or vehicle (red squares) and db/+ mice (green triangles); *n* = 10.

Db/db mice presented significantly higher blood glucose levels than nondiabetic mice (Figure 1A), confirming the diabetic status of the animals. No differences in body weight or blood glucose levels were found between diabetic mice treated with sitagliptin or vehicle eye drops (Figure 1A,B). This finding indicates that all the observed effects can only be attributed to the direct effect of the drug on the retina and not to a systemic metabolic improvement.

### 3.2. Sitagliptin Eye Drops Prevented the Antioxidant Deficiencies of the Diabetic Retina

**Figure 2:**
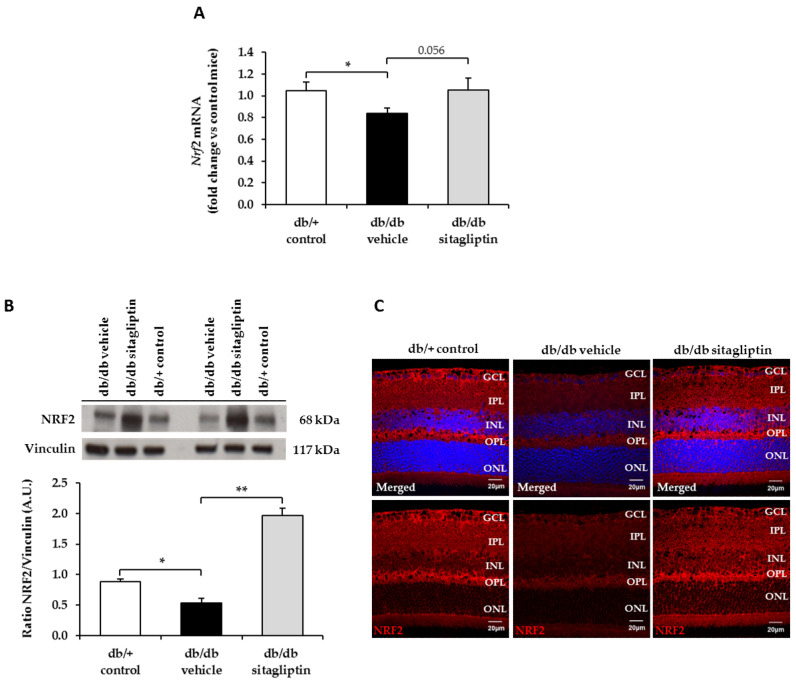
Study of the mRNA and protein levels of NRF2. (**A**) RT-PCR analysis of the gene that codifies for NRF2 (Nrf2) in db/db mice topically treated with vehicle (black bars), sitagliptin (gray bars), and in db/+ mice (white bars). Results are presented as fold change vs. control mice; *n* = 4. (**B**) Western blot bands and densitometric analysis of NRF2 protein levels in retinas of vehicle-treated db/db mice (black bars), sitagliptin-treated db/db mice (grey bars) and nondiabetic mice (white bars); *n* = 3. (**C**) Representative images of NRF2 (red) retinal immunofluorescence in diabetic mice treated with vehicle or sitagliptin eye drops and in nondiabetic mice. Hoechst staining (blue) was used for nuclei labeling. Scale bars, 20 µm; *n* = 4. GCL (ganglion cell layer), IPL (inner plexiform layer), INL (inner nuclear layer), OPL (outer plexiform layer), ONL (outer nuclear layer). * *p* < 0.05; ** *p* < 0.01.

**Figure 3:**
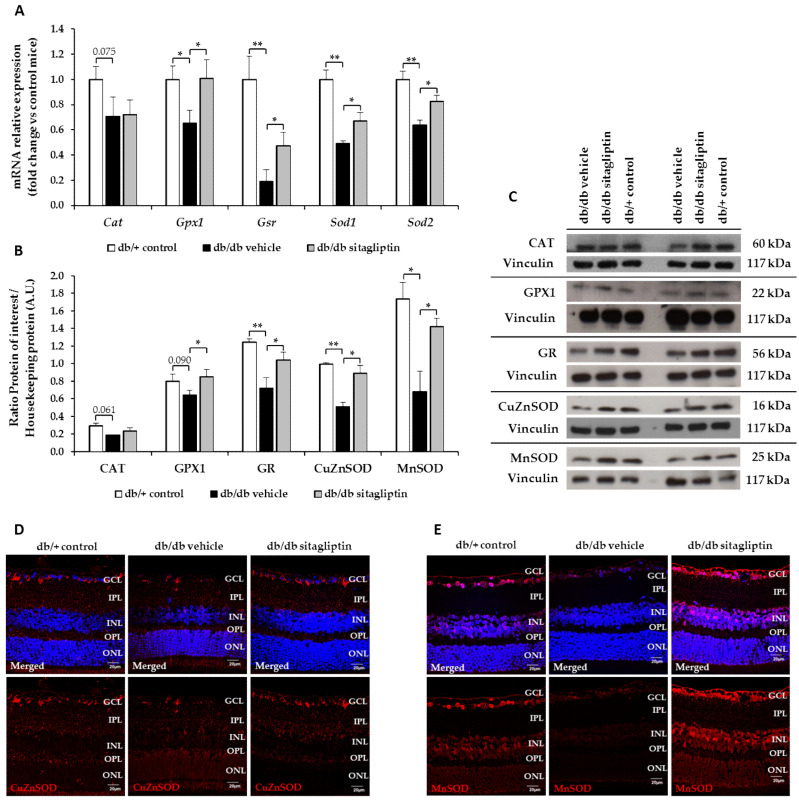
Study of the mRNA and protein levels of the most relevant antioxidant enzymes. (**A**) RT-PCR analysis of the genes that codify for CAT (Cat), GPX isoform 1 (Gpx1), GR (Gsr), CuZnSOD (Sod1), and MnSOD (Sod2) in db/db mice topically treated with vehicle (black bars) or sitagliptin (gray bars) and in db/+ mice (white bars). Results are presented as fold change vs. control mice; *n* = 4. (**B**,**C**) Densitometric analysis and Western blot bands of CAT, GPX isoform 1 (GPX1), GR, CuZnSOD and MnSOD protein levels in retinas of vehicle-treated db/db mice (black bars), sitagliptin-treated db/db mice (grey bars), and nondiabetic mice (white bars); *n* = 3. (**D**,**E**) Representative images of the retinal immunofluorescence of CuZnSOD (red) (**D**) and MnSOD (red) (**E**) in diabetic mice treated with vehicle or sitagliptin eye drops and in nondiabetic mice. Hoechst staining (blue) was used for nuclei labeling. Scale bars, 20 µm; *n* = 4. GCL (ganglion cell layer), IPL (inner plexiform layer), INL (inner nuclear layer), OPL (outer plexiform layer), ONL (outer nuclear layer). * *p* < 0.05; ** *p* < 0.01.

Nuclear factor (erythroid-derived 2)-like 2 (NRF2) is a redox-sensitive transcription factor that plays a major defensive role in the NVU by modulating the expression of antioxidant enzymes, regulating microglial dynamics, and protecting neurons and astrocytes from toxins [15]. It binds to the antioxidant response elements (ARE), which are located in the promoter region of genes that encode many antioxidant enzymes, such as copper–zinc superoxide dismutase (CuZnSOD), manganese superoxide dismutase (MnSOD), catalase (CAT), glutathione peroxidase (GPX), or glutathione reductase (GR) [16]. In db/db mice, mRNA and protein levels of NRF2 were downregulated and topical sitagliptin treatment prevented decreased protein levels in all retinal layers (Figure 2A–C). Regarding all the aforementioned antioxidant enzymes, their retinal mRNA and protein levels were also downregulated in db/db mice, while sitagliptin prevented these abnormalities with the exception of catalase, on which it exerted a neutral effect (Figure 3A–E). Topical administration of sitagliptin prevented this downregulation in all the retinal layers (i.e., MnSOD) or just in GCL (i.e., CuZnSOD) (Figure 3D,E).

**Figure 4:**
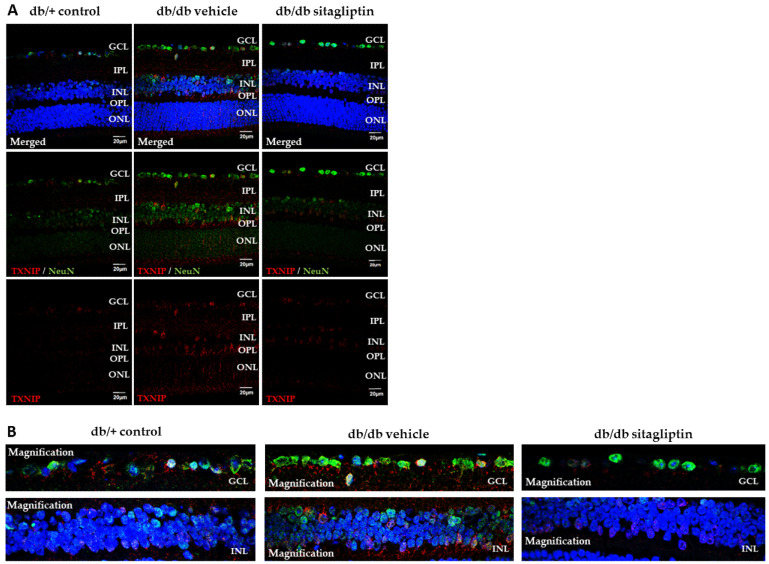
TXNIP immunofluorescence assay. (**A**,**B**) Representative images (**A**), with a magnification of the GCL and INL layers (**B**) of TXNIP (red) retinal immunofluorescence in db/db mice treated with vehicle or sitagliptin eye drops and in db/+ mice. NeuN (green) and Hoechst staining (blue) were used as neuronal and nuclei markers, respectively. Scale bars, 20 µm; *n* = 4. GCL (ganglion cell layer), IPL (inner plexiform layer), INL (inner nuclear layer), OPL (outer plexiform layer), ONL (outer nuclear layer).

NRF2 regulates the transcription of other genes whose physiological expression is altered by hyperglycemia and oxidative stress, such as the gene encoding thioredoxin interacting protein (TXNIP) [17,18]. TXNIP is a prooxidant and proapoptotic protein up-regulated in DR, which acts by inhibiting the ROS scavenging and thiol-reducing capability of the antioxidant enzyme thioredoxin (TRX). TXNIP expression has been strongly linked to hyperglycemia in retinal cell cultures, and its sustained expression over time leads to oxidative stress, inflammation, and premature cell death [18]. In db/db mice treated with vehicle, the number of TXNIP-positive cells was significantly higher than in control mice, while the values obtained in sitagliptin-treated db/db mice were similar to controls (Figure 4A,B).

### 3.3. Topical Administration of Sitagliptin Reduced the Aberrant Levels of Superoxide and Their Consequent Oxidative Damage to Biological Macromolecules in Diabetic Retinas

**Figure 5:**
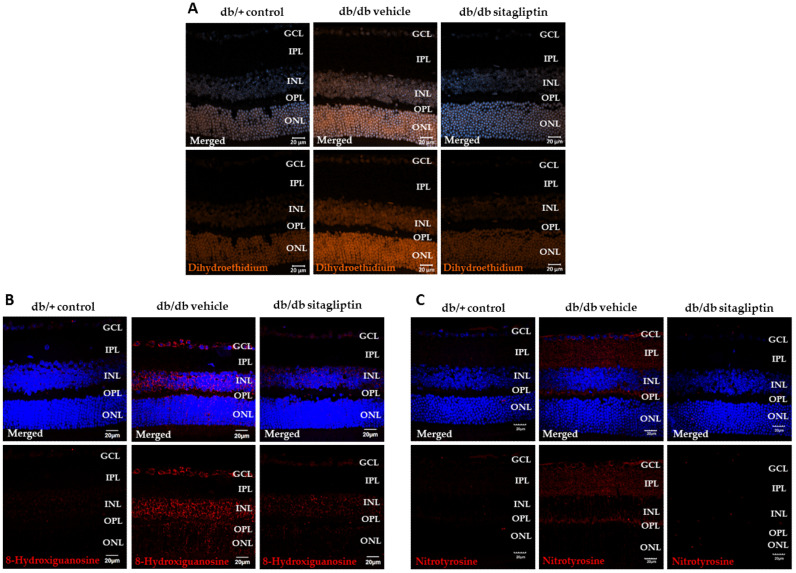
Evaluation of superoxide levels and consequent damage to biological macromolecules. (**A**–**C**) Representative images of retinal immunofluorescence of dihydroethidium (orange) (**A**), 8-hydroxiguanosine (red) (**B**), and nitrotyrosine (red); (**C**) immunostaining in db/db mice topically treated with vehicle or sitagliptin and in control mice. Hoechst staining (blue) was used for nuclei labeling. Scale bars, 20 µm; *n* = 4. GCL (ganglion cell layer), IPL (inner plexiform layer), INL (inner nuclear layer), OPL (outer plexiform layer), ONL (outer nuclear layer).

Excessive production of superoxide radicals is one of the main contributors to hyperglycemia-related oxidative stress in the diabetic retina [19]. Dihydroethidium (5-ethyl-5,6-dihydro-6-phenyl-3,8-diaminophe-nanthridine, hydroethidine, DHE) is a hydrophobic and uncharged molecule that has the capacity of crossing extra and intracellular membranes, where it can be oxidized by superoxide, giving rise to two different fluorescent products: ethidium, which is formed by specific redox reactions, and 2-hydroxyethidium, which is a specific adduct of superoxide. Therefore, DHE staining can be used to assess superoxide detection [20]. We found that relative DHE immunofluorescence expression was higher in the nuclear layers of diabetic mice than in the same layers of nondiabetic mice, and that sitagliptin reduced the hyperglycemia-related overproduction of O_2_^•^^−^ (Figure 5A).

High levels of ROS and reactive nitrogen species (RNS) facilitate their binding to biological such macromolecules as DNA, proteins, and lipids. These interactions promote damage to cell components, thus resulting in biological dysfunctions [21]. One of the most used markers is 8-hydroxyguanosine (8-OHG), the predominant product of DNA/RNA oxidative damage [22]. Nitrotyrosine is another excellent biomarker of oxidative stress, and is formed as a result of protein nitration of free tyrosine residues by reactive peroxynitrite molecules [23]. In our study, both indicators of oxidative damage were significantly higher in the nuclear layers of diabetic retinas treated with vehicle than in the same layers of nondiabetic retinas. We observed that sitagliptin significantly prevented these DR-related abnormalities (Figure 5B,C).

### 3.4. High PKC Presence in Diabetic Retinas was Reduced by Topical Treatment with Sitagliptin

**Figure 6:**
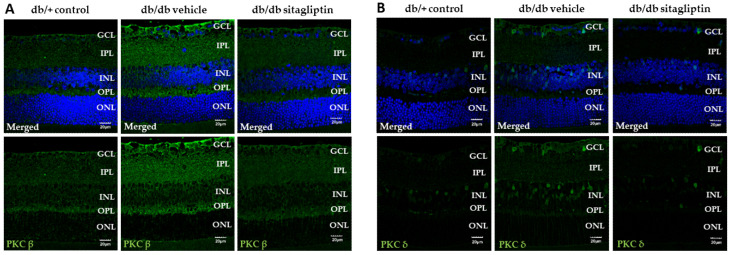
Study of PKC activation. (**A**,**B**) Representative images of the retinal immunofluorescence of PKC-β (green) (**A**) and PKC-δ (green) (**B**) in db/db mice topically treated with vehicle or sitagliptin and in db/+ mice. Hoechst staining (blue) was used for nuclei labeling. Scale bars, 20 µm; *n* = 4. GCL (ganglion cell layer), IPL (inner plexiform layer), INL (inner nuclear layer), OPL (outer plexiform layer), ONL (outer nuclear layer).

Hyperactivation of protein kinase C (PKC) isoforms is produced by oxidative stress, and, in turn, contributes to oxidative stress. The PKC family is composed of 12 isoforms, in which PKC-α, -β, -δ, and -ε activation play a key role in the development of DR [10]. In the present study, the relative immunofluorescence expression of PKC-β and the number of PKC-δ positive cells were significantly higher in db/db mice treated with vehicle than in control mice. Treatment with sitagliptin eye drops avoided this abnormal increase of both PKC isoforms (Figure 6A,B).

### 3.5. Sitagliptin Exhibited Anti-Inflammatory Properties When Administered Topically in Diabetic Retinas

**Figure 7:**
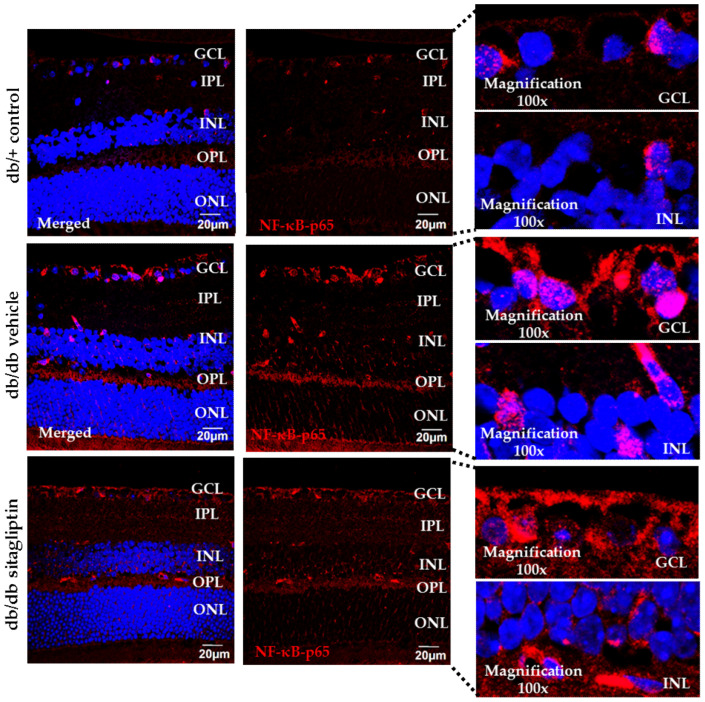
Comparison of NF-κB translocated cells (red) through immunofluorescence. NF-κB relative fluorescence intensity is displayed alone and merged with Hoechst nuclei staining (blue). A 100× magnification of merged images for each group is attached. Scale bars, 20 µm; *n*= 4. GCL (ganglion cell layer), IPL (inner plexiform layer), INL (inner nuclear layer), OPL (outer plexiform layer), ONL (outer nuclear layer).

**Figure 8:**
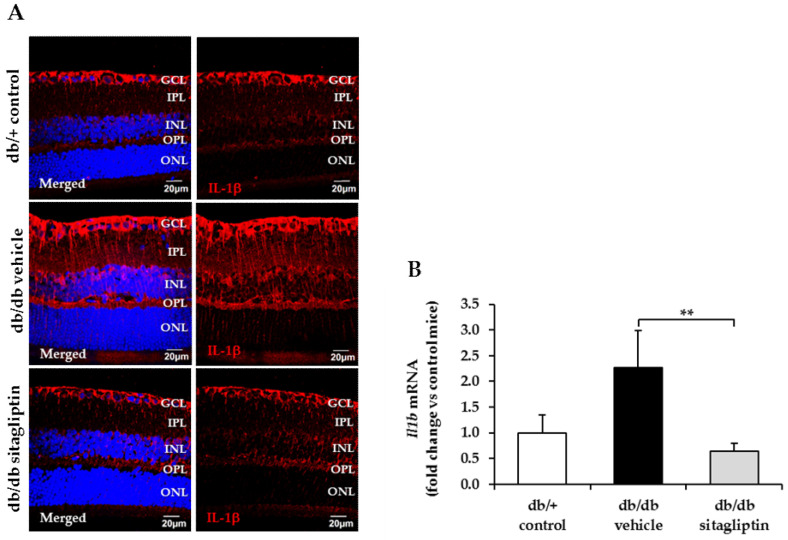
IL-1β expression. (**A**) Representative images of IL-1β protein levels (red) through immunofluorescence in db/db mice treated with vehicle or sitagliptin and in db/+ mice. Relative fluorescence intensity is displayed alone and merged with Hoechst nuclei staining (blue). Scale bars, 20 µm; *n* = 4. (**B**) Comparison of IL-1β mRNA levels. Results are presented as fold change vs. control mice. Black bar for vehicle treatment, gray bar for sitagliptin treatment, and white bar for nondiabetic mice; *n* = 4. GCL (ganglion cell layer), IPL (inner plexiform layer), INL (inner nuclear layer), OPL (outer plexiform layer), ONL (outer nuclear layer). ** *p* < 0.01.

PKC pathway activation not only increases ROS production but also promotes nuclear factor kappa-light-chain-enhancer of activated B cells (NF-κB). Inactive NF-κB is located in the cytosol of almost all cell types, forming a complex with the inhibitory protein nuclear factor of kappa light polypeptide gene enhancer in B-cells inhibitor alpha (IκBα). Several mechanisms, including hyperglycemia or oxidized proteins, can activate the enzyme IκB kinase (IKK) which phosphorylates IκBα. As result of phosphorylation, IκBα is ubiquitinated, dissociated from the NF-κB complex, and finally degraded by the proteasome. This signaling pathway provokes the activation of NF-κB and its consequent translocation to the cell nucleus, where it regulates inflammatory responses (i.e., cytokine production) [24]. Regarding our study, topical ocular administration of sitagliptin in db/db mice reduced NF-κB translocation in comparison to vehicle-treated db/db mice, showing similar results to the nondiabetic condition (Figure 7). Furthermore, we observed an increase in mRNA and protein levels of interleukin 1 beta (IL-1β) in vehicle-treated diabetic mice, which was attenuated by sitagliptin (Figure 8A,B).

## 4. Discussion

In the present study, we demonstrated the antioxidant and the anti-inflammatory properties of sitagliptin, a DPP-4i, in an experimental model of DR. These findings support and reinforce previous evidence showing the beneficial effects of topical administration of sitagliptin in early stages of DR [12,13].

Hyperglycemia-induced oxidative stress is one of the greatest threats to the retinal NVU. The oxygen consumption rate of the retina is extremely high, and it has an abundance of polyunsaturated acids, which are very susceptible to lipid peroxidation [9]. A clear relationship between the diabetic milieu and both ROS overproduction and deficiencies in the antioxidant machinery has been reported in multiple studies [25]. The physiological sources of ROS, mainly the mitochondrial electron transport chain (ETC) and the nicotinamide adenine dinucleotide (NAD^+^/NADH) phosphate (NADPH) oxidase family of enzymes (NOX), are disrupted in DR, leading to an excessive generation of ROS [26]. By contrast, the activity of antioxidant enzymes, which are directly or indirectly responsible for ROS and RNS scavenging, and the transcriptional functionality of the antioxidant factor NRF2 are both diminished in DR [27]. NRF2 is a major regulator of redox homeostasis, with an important role as a negative regulator of inflammation, attenuating oxidative stress by scavenging reactive oxygen species (ROS) and preventing genomic instability due to DNA damage. In the present study, we provide evidence that in db/db mice there exists a significant increase in superoxide levels and oxidative damage to DNA/RNA and proteins and a notable downregulation of NRF2 and the antioxidant enzymes CAT, GPX, GR, CuZnSOD, and MnSOD in comparison with db/+ nondiabetic control mice.

The capacity of sitagliptin to activate the NRF2/ARE pathway and ameliorate the pathological outcome of diseases where oxidative stress plays a key role has already been evidenced. For example, in a β-amyloid-induced rat model of Alzheimer’s disease, sitagliptin improved cognitive status by its antioxidant effects mediated by the activation of NRF2 [28]. Sitagliptin was also able to increase the activities of some antioxidant enzymes, such as SOD, independently of their glucose-lowering abilities [29]. In the present study, we found that topical (eye drops) administration of sitagliptin modulated positively mRNA and protein production by the murine retina of both antioxidant elements: the NRF2/ARE pathway and the enzymatic defenses GPX, GR, CuZnSOD, and MnSOD. In addition, we found that sitagliptin prevented the upregulation of TXNIP (a prooxidant and proapoptotic protein) induced by diabetes, which plays a relevant role in the pathogenesis of DR [18]. Remarkably, we further detected that the topical administration of sitagliptin also reduced the RNA/DNA oxidative damage induced by RNS and ROS in the retinas of db/db mice.

It should be emphasized that the absence of differences in blood glucose levels between diabetic mice treated with vehicle or with sitagliptin eye drops support the idea that sitagliptin has antioxidant properties that cannot be attributed to its glucose-lowering capacity. Although there is some information regarding the antioxidant effect of sitagliptin when administered systemically [30,31], it is impossible to know whether this effect is due to the lowering effect of blood glucose levels or a direct effect of sitagliptin. In addition, the second possibility is unlikely, because it has been reported that sitagliptin in unable to cross the blood–retina barrier. Taken together, to the best of our knowledge, this is the first evidence of direct antioxidant effects of sitagliptin in the diabetic retina after its topical ocular administration.

Little is known about how DPP-4i exert their antioxidant and intrinsic functions. Since the increase in GLP-1 availability is one of the main consequences of DPP-4i administration, it could be postulated that GLP-1, rather than DPP4i, exerts the antioxidant effect. In fact, in previous studies we demonstrated that topically administered GLP-1 also has the capability of reducing oxidative damage in the same experimental model of DR [32]. However, the antioxidant effects of DPP4i have also been observed in in vitro models without GLP-1 [33]. In addition, we have found that sitagliptin, but not GLP-1 eye drops, improved the deficiencies in GPX and GR protein expressions and prevented the downregulation of CuZnSOD levels in the GCL layer. These findings suggest that sitagliptin may have GLP-1-independent mechanisms of action. These GLP-1-independent beneficial properties together with GLP-1-mediated effects, the lower price, and higher stability in comparison with GLP-1, make sitagliptin eye drops an ideal candidate to be tested in clinical trials for treating early stages of DR.

One of the biggest obstacles in the study of retinal NVU impairment in the context of early stages of DR is to establish a chronological order between the different disrupters (i.e., inflammation, glial activation, oxidative stress, neurodegeneration, and vascular leakage). PKC hyperactivation is one of the mechanisms that has been postulated as a possible link between hyperglycemia and oxidative stress in DR [34]. It is not still clear whether PKC activation contributes more to oxidative stress than oxidative stress contributes to its enhancement [10]. Geraldes et al. [35] reported that hyperglycemia promotes through PKC-δ two different fundamental pathways: cumulative ROS production and NF-κB activation. Notably, we have found that sitagliptin eye drops prevented the diabetes-induced upregulation of PKC-β and PKC-δ.

Hyperglycemia-induced oxidative stress leads to other pathological events, such as inflammation [36]. In the present study, we observed that topical administration of sitagliptin reduced NF-κB translocation and the production of IL-1β, proving the anti-inflammatory properties of DPP-4i. Likewise, Li et al. [37] demonstrated that linagliptin, another DPP-4i, reduced TNF-α accumulation and NF-κB activation in retinal endothelial cells. Gonçalves et al. [30] also proved sitagliptin’s capability to reduce nitrosative stress and IL-1β in the retinas of Zucker Diabetic Fatty (ZDF) rats. However, in this case, since sitagliptin was administered by the oral route, the beneficial effects could be attributed to the improvement in blood glucose control.

As a limiting factor, it could be argued that the duration of treatment (2 weeks) was too short. However, all the papers testing the effectiveness of DPP-IV inhibitors and other molecules addressed to treat early stages of DR using topical administration have been performed using similar treatment duration [12,38,39,40,41,42,43]. It should be noted that we are treating early stages of DR in which microvascular retinal lesions are still absent on fundoscopic examination. However, neurodegeneration and impairment of the neurovascular unit could already be present. These abnormalities can occur very shortly after the onset of diabetes, and the time needed for any treatment to prevent or arrest them in the clinical arena remains to be elucidated. Nevertheless, although we are assuming that the effect of sitagliptin would persist if the treatment were longer, specific studies addressed to confirm this assumption are needed.

## 5. Conclusions

Although a close relationship between the diabetic milieu and both ROS overproduction and deficiencies in the antioxidant machinery have been reported in multiple studies, the underlying mechanisms involved in diabetes-induced oxidative stress remain to be elucidated. Our results suggest that the db/db mouse model recapitulates well the imbalance between ROS production and antioxidant defenses that exists in the diabetic retina. In addition, we provide the first evidence of the effectiveness of topical (eye drops) administration of sitagliptin in preventing diabetes-induced oxidative stress in the retina. This finding, together our previous reports showing neurovascular protection of sitagliptin, suggest this therapeutic strategy is promising, and therefore specific clinical trials seem warranted.

## Figures and Tables

**Table 1:** Primers used for RT-PCR experiments.

Primers	Gene ID		Nucleotide Sequence
*Actb*	11461	Forward (5′-3′)	5′-CTAAGGCCAACCGTGAAAG -3′
		Reverse (5′-3′)	5′-CAGTATGTTCGGCTTCCCATTC-3′
*B2m*	12010	Forward (5′-3′)	5′-GTATGCTATCCAGAAAACCC-3′
		Reverse (5′-3′)	5′-CTGAAGGACATATCTGACATC-3′
*Cat*	12359	Forward (5′-3′)	5′-AGCGACCAGATGAAGCAGTG-3′
		Reverse (5′-3′)	5′-TCCGCTCTCTGTCAAAGTGTG-3′
*Gpx1*	14775	Forward (5′-3′)	5′-CTCACCCGCTCTTTACCTTCCT-3′
		Reverse (5′-3′)	5′-ACACCGGAGACCAAATGATGTACT-3′
*Gsr*	14782	Forward (5′-3′)	5′-GACACCTCTTCCTTCGACTACC-3′
		Reverse (5′-3′)	5′-CCCAGCTTGTGACTCTCCAC-3′
*Il1b*	16176	Forward (5′-3′)	5′-GCAACTGTTCCTGAACTCAACT-3′
		Reverse (5′-3′)	5′-ATCTTTTGGGGTCCGTCAACT-3′
*Nrf2*	18024	Forward (5′-3′)	5′-TCTTGGAGTAAGTCGAGAAGTGT-3′
		Reverse (5′-3′)	5′-GTTGAAACTGAGCGAAAAAGGC-3′
*Sod1*	20655	Forward (5′-3′)	5′-AACCAGTTGTGTTGTCAGGAC-3′
		Reverse (5′-3′)	5′-CCACCATGTTTCTTAGAGTGAGG-3′
*Sod2*	20656	Forward (5′-3′)	5′-CAGACCTGCCTTACGACTATG-3′
		Reverse (5′-3′)	5′-CTCGGTGGCGTTGAGATTGTT-3′

**Table 2:** Primary antibodies used for Western blot experiments.

Primary Antibodies	Description
Catalase	Rabbit polyclonal; 1:1000; GTX110704; GeneTex, Alton Pkwy Irvine, CA, USA
Copper-zinc superoxide dismutase	Rabbit polyclonal; 1:1000; GTX100554; GeneTex, Alton Pkwy Irvine, CA, USA
Glutathione peroxidase 1	Rabbit polyclonal; 1:1000; 55053-1-AP; Proteintech, Rosemont, IL, USA
Glutathione reductase	Mouse monoclonal; 1:1000; sc-133245; Santa Cruz, Dallas, TX, USA
Manganese superoxide dismutase	Rabbit polyclonal; 1:1000; ab13533; Abcam, Cambridge, UK
NRF2	Rabbit polyclonal; 1:1000; 16396-1-AP; Proteintech, Rosemont, IL, USA
Vinculin	Mouse monoclonal; 1:7000; sc-73614; Santa Cruz, Dallas, TX, USA

**Table 3:** Primary and secondary antibodies used for immunofluorescence experiments.

Primary Antibodies	Description
8-Hydroxyguanosine	Mouse monoclonal; 1:100; ab62623; Abcam, Cambridge, UK
Collagen IV	Rabbit monoclonal; 1:200; ab236640; Abcam, Cambridge, UK
Copper-zinc superoxide dismutase	Rabbit polyclonal; 1:100; GTX100554; GeneTex, Alton Pkwy Irvine, CA, USA
Dihydroethidium	D23107; ThermoFisher Scientific, Waltham, MA, USA
IL-1β	Rabbit polyclonal; 1:200; ab9722; Abcam, Cambridge, UK
Ki67	Rabbit polyclonal; 1:500; ab15580; Abcam, Cambridge, UK
Manganese superoxide dismutase	Rabbit polyclonal; 1:100; ab13533; Abcam, Cambridge, UK
NeuN	Mouse monoclonal; 1:200; ab104224; Abcam, Cambridge, UK
NF-кB	Mouse monoclonal; 1:100; sc-8008; Santa Cruz, Dallas, TX, USA
Nitrotyrosine	Mouse monoclonal; 1:100; ab7048; Abcam, Cambridge, UK
NRF2	Rabbit polyclonal; 1:1000; 16396-1-AP; Proteintech, Rosemont, IL, USA
Protein kinase C-β	Rabbit polyclonal; 1:100; ab189782; Abcam, Cambridge, UK
Protein kinase C-δ	Rabbit monoclonal; 1:100; ab182126; Abcam, Cambridge, UK
TXNIP	Rabbit monoclonal; 1:200; ab188865; Abcam, Cambridge, UK
**Secondary antibodies**	**Description**
Alexa Fluor 488 Goat anti-mouse	Goat polyclonal; 1:600; ab150113; Abcam, Cambridge, UK
Alexa Fluor 488 Goat anti-rabbit	Goat polyclonal; 1:600; ab150081; Abcam, Cambridge, UK
Alexa Fluor 594 Goat anti-mouse	Goat polyclonal; 1:600; A-11032; ThermoFisher Scientific, Waltham, MA, USA
Alexa Fluor 594 Goat anti-rabbit	Goat polyclonal; 1:600; A-11012; ThermoFisher Scientific, Waltham, MA, USA

## Data Availability

The data presented in this study are available in the article.
